# LGR5 is Expressed by Ewing Sarcoma and Potentiates Wnt/β-Catenin Signaling

**DOI:** 10.3389/fonc.2013.00081

**Published:** 2013-04-15

**Authors:** Christopher A. Scannell, Elisabeth A. Pedersen, Jack T. Mosher, Melanie Anne Krook, Lauren A. Nicholls, Breelyn A. Wilky, David M. Loeb, Elizabeth R. Lawlor

**Affiliations:** ^1^Keck School of Medicine, University Southern CaliforniaLos Angeles, CA, USA; ^2^Department of Pediatrics, University of MichiganAnn Arbor, MI, USA; ^3^Department of Medical Oncology, Sidney Kimmel Comprehensive Cancer Center, Johns Hopkins UniversityBaltimore, MD, USA; ^4^Division of Pediatric Oncology, Sidney Kimmel Comprehensive Cancer Center, Johns Hopkins UniversityBaltimore, MD, USA; ^5^Department of Pathology, University of MichiganAnn Arbor, MI, USA

**Keywords:** LGR5, Ewing sarcoma, stem cell, R-spondin, Wnt, β-catenin

## Abstract

Ewing sarcoma (ES) is an aggressive bone and soft tissue tumor of putative stem cell origin that predominantly occurs in children and young adults. Although most patients with localized ES can be cured with intensive therapy, the clinical course is variable and up to one third of patients relapse following initial remission. Unfortunately, little is yet known about the biologic features that distinguish low-risk from high-risk disease or the mechanisms of ES disease progression. Recent reports have suggested that putative cancer stem cells exist in ES and may contribute to an aggressive phenotype. The cell surface receptor leucine-rich repeat-containing G-protein coupled receptor 5 (LGR5) is a somatic stem cell marker that functions as an oncogene in several human cancers, most notably colorectal carcinoma. LGR5 is a receptor for the R-spondin (RSPO) family of ligands and RSPO-mediated activation of LGR5 potentiates Wnt/β-catenin signaling, contributing to stem cell proliferation and self-renewal. Given its presumed stem cell origin, we investigated whether LGR5 contributes to ES pathogenesis. We found that *LGR5* is expressed by ES and that its expression is relatively increased in cells and tumors that display a more aggressive phenotype. In particular, *LGR5* expression was increased in putative cancer stem cells. We also found that neural crest-derived stem cells express *LGR5*, raising the possibility that expression of *LGR5* may be a feature of ES cells of origin. *LGR5-*high ES cells showed nuclear localization of β-catenin and robust activation of TCF reporter activity when exposed to Wnt ligand and this was potentiated by RSPO. However, modulation of *LGR5* or exposure to RSPO had no impact on proliferation confirming that Wnt/β-catenin signaling in ES cells does not recapitulate signaling in epithelial cells. Together these studies show that the RSPO-LGR5-Wnt-β-catenin axis is present and active in ES and may contribute to tumor pathogenesis.

## Introduction

Ewing sarcoma (ES) is a malignant tumor of the bone and soft tissue that can present at any age but predominantly occurs in adolescents and young adults. These tumors are genetically defined by recurrent chromosomal translocations that result in the creation of novel fusion oncogenes, most commonly *EWS-FLI1* (Balamuth and Womer, 2010). Clinically, these tumors often have an aggressive course with one quarter of patients presenting with gross metastatic disease at the time of diagnosis. In addition, nearly one third of patients will relapse after an initial clinical remission and patients who have metastatic or relapsed ES have 5-year event free survival rates of only 10–20% (Balamuth and Womer, 2010). Unfortunately, there are no clinical or pathologic criteria apart from metastases that can reliably predict whether a newly diagnosed patient with ES is likely to be cured or to relapse. Histologically, tumors are characterized by an undifferentiated small round blue cell phenotype with features of primitive neuroectodermal cells. Although predominantly a bone and connective tissue tumor, clinically ES can present in multiple organs and tissue types throughout the body, suggesting a relatively undifferentiated and potentially highly migratory cell of origin (Meltzer, 2007). Indeed, current evidence supports the hypothesis that ES arise from either mesenchymal stem cells (MSC) or neural crest stem cells (NCSC) or their early progenitors (Staege et al., 2004; Tirode et al., 2007; Riggi et al., 2008; von Levetzow et al., 2011). Importantly, poorly differentiated tumors in other classes of human malignancy often express stem cell-associated markers and an undifferentiated phenotype combined with high-level expression of stem cell genes is associated with worse clinical outcomes (Phillips et al., 2006; Ben-Porath et al., 2008; Spike et al., 2012). The stem cell phenotype and aggressive nature of ES raise the question of whether stem cell markers could be useful in understanding the origin and pathogenesis of this enigmatic disease.

Leucine-rich repeat-containing G-protein coupled receptor 5 (LGR5) is a seven transmembrane spanning receptor that has recently been identified as a somatic stem cell marker that plays key functional roles in both normal development and cancer. Mouse studies have demonstrated that *Lgr5* is widely expressed during embryonic development but expression in postnatal tissues is limited to discrete stem cell populations (Barker et al., 2007). Such stem cells can be found in the small and large intestine, stomach, hair follicles, and kidney (Barker et al., 2007, 2010, 2012; Jaks et al., 2008). The self-renewal and proliferation of normal murine intestinal stem cells (ISC) is dependent on Lgr5 (Barker et al., 2007). Significantly, Lgr5^+^ ISC have also recently been identified as both the cells of origin for murine intestinal tumors and tumor-maintaining cancer stem cells in established adenomas (Barker et al., 2009; Schepers et al., 2012). Studies of human cancer cell lines have now confirmed that *LGR5* promotes the growth and/or survival of colorectal and basal cell carcinoma (McClanahan et al., 2006; Tanese et al., 2008), glioblastoma (Nakata et al., 2013), and neuroblastoma (Balamuth et al., 2010). Thus, LGR5 has now been shown to contribute functionally to normal and malignant biology in tissues of both epithelial and neural origin. To determine if high levels of *LGR5* are associated with a more aggressive clinical course, retrospective studies of archived tumors were undertaken and showed diminished survival in gastrointestinal carcinoma and glioblastoma patients whose tumors expressed high levels of *LGR5* (Becker et al., 2010; Wu et al., 2012; Nakata et al., 2013). In addition, in the case of colorectal carcinoma, LGR5 is expressed by a subpopulation of cells with stem cell-like properties (i.e., cancer stem cells or CSC) (Kemper et al., 2012; Kobayashi et al., 2012). The LGR5^+^ colorectal CSC have increased clonogenic and tumorigenic potential compared to bulk tumor cells and lose expression of LGR5 upon *in vitro* differentiation (Kemper et al., 2012). Gene expression profiling experiments have also demonstrated that an Lgr5-stem cell gene signature predicts disease relapse in colorectal cancer patients (Merlos-Suarez et al., 2011). Thus, there is compelling evidence in both human and murine intestinal tumors that LGR5^+^ stem cells contribute to cancer initiation and progression and that high *LGR5* expression is associated with worse clinical outcomes.

The mechanism by which LGR5 promotes stem cell self-renewal and proliferation has only recently been elucidated. It is now known that, along with its closely related homologs LGR4 and LGR6, LGR5 functions to potentiate canonical Wnt/β-catenin signaling (Carmon et al., 2011; de Lau et al., 2011; Glinka et al., 2011; Gong et al., 2012). This potentiation of Wnt signaling is achieved when LGR5 is bound by its ligand R-spondin (RSPO). RSPOs are a recently described family of secreted proteins that function as Wnt agonists and play pivotal roles as regulators of normal embryonic development and stem cell proliferation (Glinka et al., 2011). When RSPO binds to LGR5 the receptor associates with the Frizzled/LRP complex to increase the activation of β-catenin and downstream TCF reporter activity as well as non-canonical Wnt signaling (Glinka et al., 2011). The potentiation of Wnt/β-catenin signaling is now believed to mediate the self-renewal and proliferation of LGR5^+^ stem cells, both normal and malignant, and provides a mechanistic link between LGR5, Wnt signaling, cancer stem cells, and cancer progression (Clevers and Nusse, 2012).

Given the presumed stem cell origin of ES, the presence of RSPO2 in developing bone (Friedman et al., 2009) and prior evidence that Wnt signaling is abnormal in these tumors (Uren et al., 2004; Navarro et al., 2010; Vijayakumar et al., 2011) we hypothesized that *LGR5* and its downstream impact on Wnt signaling might contribute to ES pathogenesis. In the current study we have investigated this hypothesis using studies of ES primary tumors and cell lines and normal neuro-MSC, candidate cells of ES origin. Our findings indicate that *LGR5* is indeed expressed by some populations of ES cells and that these cells upregulate Wnt/β-catenin signaling when exposed to RSPO. Together our studies suggest that LGR5-mediated potentiation of Wnt signaling may be an important contributor to ES initiation and maintenance, especially in RSPO-rich microenvironments like developing bone.

## Materials and Methods

### Tumor samples and cell lines

Primary tumor RNA was obtained from Children’s Hospital Los Angeles (CHLA) and Children’s Oncology Group (COG) tumor biorepositories. All primary tissue samples were coded according to an anonymous numbering scheme and acquired in accordance with approval from the CHLA Committee for Clinical Investigation. Primary tumor expression data were kindly provided by the COG (Lawlor, unpublished data). ES cell lines were kindly provided by Dr. Timothy Triche (CHLA, Los Angeles, CA, USA), Dr. Heinrich Kovar (CCRI, St. Anna Kinderkrebsforschung, Vienna, Austria), and the COG cell bank (cogcell.org) and identities confirmed by short tandem repeat profiling courtesy of Dr. Pat Reynolds (Texas Tech University, Lubbock, TX, USA). All ES cell lines were maintained in RPMI with l-glutamine and 10% FBS on tissue culture treated polystyrene plates, except for CHLA25 and STA-ET-8.2 cells. These cell lines were grown on plates coated with fibronectin. Human bone marrow mesenchymal stem cell lines (MSC; obtained from Dr. Darwin Prockop, Tulane University, New Orleans, LA, USA), H1 and H9 human embryonic stem cell (ESC) lines (Wicell, Madison, WI, USA) and ESC-derived neural crest cells were cultured and differentiated using standard protocols as previously described by our lab (Jiang et al., 2009; von Levetzow et al., 2011). MRC5 fibroblasts were obtained from American Type Culture Collection and maintained in DMEM with l-glutamine and 10% FBS.

### Quantitative real-time reverse transcription PCR

cDNA was generated from RNA (iScript; Bio-Rad, Hercules, CA, USA) and qRT-PCR was performed with validated TaqMan primers (*LGR5*, *CITED2*, *CYCLIND1*, *MYC*, *GAPDH*, and *18S*; Life Technologies, Grand Island, NY, USA) or primers designed using qPrimerDepot (primerdepot.nci.nih.gov) (Table 1) (Cui et al., 2007). Assays were performed in triplicate using the Applied Biosystems 7900HT Fast real-time PCR system or Roche Light Cycler 480 and average *Ct* values were normalized relative to *GAPDH* or *18S* expression in the same sample.

**Table 1:** **Prime sequences for RT-PCR**.

Gene	Forward primer sequence	Reverse primer sequence
*AXIN2*	AAGTGCAAACTTTCGCCAAC	ACAGGATCGCTCCTCTTGAA
*CD44*	GACAAGTTTTGGTGGCACG	CACGTGGAATACACCTGCAA
*CDC25A*	CCAGCCCCAAAGAGTCAAC	AAGGTCCCTTGGGTCATTGT
*EPHB2*	CTCTACTGTAACGGGGACGG	CCTTGAAAGTCCCAGATGGA
*HIG2*	CTTCTGCGCTGGTGCTTAGT	GCAGAGAAACAGAGCTGCCT
*ID2*	GACAGCAAAGCACTGTGTGG	TCAGCACTTAAAAGATTCCGTG
*LEF1*	TGGATCTCTTTCTCCACCCA	CACTGTAAGTGATGAGGGGG
*TROY*	CCCTCCTCCTCCTTACGAAC	CCAGAGCGCTGCAGATAAC
*GAPDH*	AAGGTGAAGGTCGGAGTCAA	AATGAAGGGGTCATTGATGG
*18S*	GCAATTATTCCCCATGAACGA	GGCCTCACTAAACCATCCAAT

### Immunocytochemistry

Cells were grown on fibronectin-coated chamber slides (Thermo Fisher Scientific, Waltham, MA, USA), fixed in 4% paraformaldehyde, and permeabilized with 0.1% Triton X-100. After rinsing in phosphate-buffered saline (PBS) the slides were incubated for 1 h in blocking solution (1% BSA and 5% donkey serum in PBS). Blocked slides were incubated with primary β-catenin antibody (1:300; BD Biosciences, San Jose, CA, USA) overnight at 4°C, washed in PBS, incubated with Alexa Fluor 488-conjugated, donkey-anti mouse secondary antibody (1:500; Life Technologies) for 1 h, and then visualized with a Leica DMI6000 B Fluorescence Microscope. Nuclei were counterstained with 4′,6-diamidino-2-phenylindole (DAPI). Cytoplasmic and nuclear β-catenin staining cells were manually counted in a minimum of three high-power fields.

### Cell sorting for subpopulation studies

For CD133 sorts, cultured STA-ET-8.2 cells were trypsinized and resuspended in FcR Blocking Reagent (Miltenyi Biotec, Auburn, CA, USA) with 0.5% bovine serum albumin (Sigma, St Louis, MO, USA) in PBS. Mouse anti-human CD133/2-PE (Miltenyi) monoclonal antibody was then added (1:11 dilution) and incubated for 15 min at 4°C in the dark. After two washes, cells were sorted on a MoFlo Astrios instrument (Beckman Coulter) at the University of Michigan Flow Cytometry Core. Positive and negative gates were determined using IgG stained and unstained controls. TC71 and MHH-ES cells were sorted on the basis of aldehyde dehydrogenase activity using the Aldefluor^®^ assay (Stem Cell Technologies, Vancouver, BC, USA). Cells were analyzed and sorted as previously described (Awad et al., 2010).

### Generation of stable knockdown and over-expression cell lines

For knockdown studies, cell lines were transduced with pLKO.1-puro lentiviral short hairpin RNAs vectors targeting *LGR5* expression (shLGR5#1-TAAGTGCCAGAACTGCTATGG and shLGR5#3-TTGTCCAAATTGCAGTAGAGC; Thermo Fisher Scientific). Control cells were transduced with a non-silencing small hairpin RNA vector containing an inert sequence (shNS-CAACAAGATGAAGAGCACCAA). Cells were selected in puromycin (1–2 μg/mL) for at least 48 h before use in subsequent experiments. For over-expression studies, cells were transduced with a lentiviral pCL6 expression vector into which the full length open reading frame for *LGR5* cDNA (NP_003658; Thermo Scientific, Clone ID BC09624) was cloned. The pCL6 vector is a modified version of the pCL1 vector including an internal ribosome entry site and woodchuck hepatitis virus post-transcription regulatory element up- and downstream, respectively, of the EGFP reporter gene (Feldhahn et al., 2007; von Levetzow et al., 2011). Control cells were transduced with empty vector that lacked the *LGR5* sequence (Empty). Cells were selected by FACS on the basis of GFP expression. Lentiviral supernatant for each construct was generated from plasmid-transfected 293FT packaging cells as previously described (von Levetzow et al., 2011).

### TCF reporter assay

To assess the transcriptional activity of the Wnt/β-catenin axis we used previously characterized TCF-promoter luciferase-reporter constructs (Fuerer and Nusse, 2010). The 7xTcf-FFluc//SV40-mCherry (p7TFC) and 7xTcf-FFluc//SV40-PuroR (p7TFP) plasmids were purchased from Addgene (plasmids 24,307 and 24,308, respectively). Cells were sorted for mCherry expression (p7TFC) by FACS or cultured with puromycin (p7TFP) to select for cells that were successfully transduced. To assess the effects of exogenous Wnt ligand on ES cells we collected conditioned media (CM) from control mouse fibroblasts (L-cells) or L-cells that have been engineered to over-express Wnt3a (L-Wnt3a-cells) obtained from the American Type Culture Collection (ATCC CRL-2648 and CRL-2647, respectively). The final dilution of L-cell CM or Wnt3a CM was 1:2 in RPMI containing 5% FBS. Recombinant human RSPO2 (R&D Systems, Minneapolis, MN, USA) was added to L-cell CM or Wnt3a CM at a concentration of 20 ng/mL. p7TFC and p7TFP-transduced cells were plated at a density of 4.0 × 10^4^ cells/well on 96-well plates and allowed to attach overnight. The cells were cultured for another 24 h in standard RPMI with 10% FBS or treated with L-cell CM or Wnt3a CM. Luciferase measurements were carried out using the Luciferase Assay System (Promega) according to the manufacturer’s protocol and read on a Molecular Devices LMAX plate reader. Measurements were normalized to mCherry mean fluorescent intensity for cells transduced with the p7TFC vector, otherwise TCF reporter activity was expressed relative to the L-cell CM control.

### Cell proliferation assays

Cell growth was assessed using the CellTiter 96 AQueous One Solution Cell Proliferation Assay (Promega, Madison, WI, USA). Cells were plated at a density of 5 × 10^3^ cells/well in 8-well replicates on 96-well flat-bottomed plates, allowed to attach overnight, and growth was assessed on Days 0, 2, and 4 post-attachment. For cells grown in the presence of exogenous Wnt ligands, WNT3A (R&D Systems), and/or RSPO2 (R&D Systems) were supplemented on Days 0 and 3 at 500 and 20 ng/mL, respectively. The normalized cell index was calculated relative to the Day 0 absorbance reading minus background.

### Statistical analysis

Unless otherwise indicated data from all experiments are expressed as mean ± SEM from a minimum of three independent experiments. The data was analyzed using GraphPad Prism software by a Student’s *t*-test and a *p* value of <0.05 was considered significant.

## Results

### *LGR5* is expressed by Ewing sarcoma and neural crest-derived stem cells

**Figure 1:**
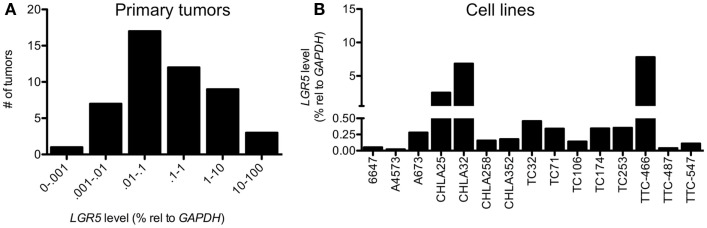
**Leucine-rich repeat-containing G-protein coupled receptor 5 is expressed by Ewing sarcomas**. qRT-PCR analysis revealed low to very high-level expression of *LGR5* in **(A)** 49 primary Ewing sarcoma (ES) and **(B)** 15 ES cell lines. For primary tumors levels of expression relative to GAPDH were <0.001% in 1 tumor, 0.001–0.01% in 7 tumors, 0.01–0.1% in 17 tumors, 0.1–1% in 12 tumors, 1–10% in 9 tumors, and 10–100% in 3 tumors.

Ewing sarcoma are usually highly undifferentiated tumors that display features of both NCSC and MSC stem cells. Thus, although their precise cellular ontogeny remains to be fully elucidated, ES are presumed to arise from malignant transformation of normal NCSC and/or MSC. We therefore sought to determine if *LGR5* is expressed by ES and the stem cells from which they are thought to arise. To address this we analyzed the expression of *LGR5* in a cohort of 49 primary ES tumor samples and 15 ES cell lines. Unfortunately, the specificity of commercially available antibodies for studies of human LGR5 remains inadequate for immuno-histochemical studies so we limited our evaluation of *LGR5* expression to quantitative RT-PCR analysis. As shown, *LGR5* was widely detectable in both ES tumors and cell lines, however, the level of expression was highly variable (Figures 1A,B).

**Figure 2:**
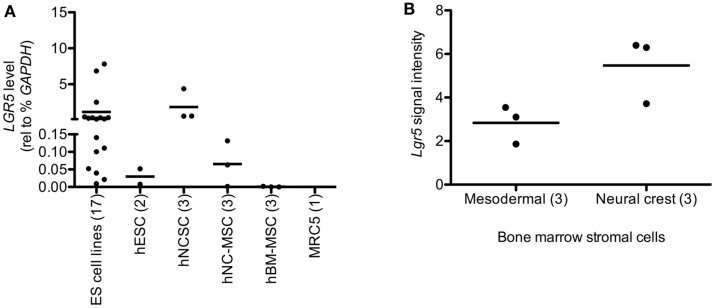
**Leucine-rich repeat-containing G-protein coupled receptor 5 is expressed by neural crest stem cells (NCSC)**. **(A)** qRT-PCR analysis of human embryonic stem cells (hESC), hESC-derived NCSC (hNCSC), hNCSC-derived mesenchymal stem cells (hNC-MSC), bone marrow-derived mesenchymal stem cells (hBM-MSC), and human lung embryo fibroblasts (MRC5) showed that undifferentiated hNCSC express the highest levels of *LGR5*. **(B)** Murine bone marrow stromal cells of neural crest origin express higher levels of *Lgr5* than cells of mesodermal origin (from publically available microarray data GEO accession GSE30419) (Wislet-Gendebien et al., 2012). *N* = 3 ± SEM. Lines represent arithmetic means of replicate samples.

Given its designation as a stem cell marker in epithelial tissues we next assessed whether *LGR5* might be expressed by NCSC and/or MSC. To address this we evaluated adult human bone marrow-derived MSC as well as human ESC-derived NCSC and their MSC progeny, neural crest-derived MSC (NC-MSC) as previously described by our group (von Levetzow et al., 2011). Significantly, our analyses revealed that undifferentiated NCSC express relatively high levels of *LGR5* whereas the transcript is undetectable in adult bone marrow-derived MSC (Figure 2A). Consistent with this observation, LGR5 expression declined when NCSC were differentiated toward an MSC identity (NC-MSC) (Figure 2A). Interestingly, the level of *LGR5* expression in these neural crest-derived stem cells was comparable to that of ES cell lines (Figure 2A). To determine if the observed differential expression of *LGR5* by neural crest cells was merely an artifact of the human ESC culture system we next interrogated publicly available gene expression data that compared murine bone marrow stem cells of mesodermal origin to bone marrow stem cells of neural crest origin (GEO accession 30,419) (Wislet-Gendebien et al., 2012). Interestingly, consistent with our human cell studies, murine bone marrow-derived stem cells of neural crest origin expressed higher levels of *Lgr5* than MSC of mesodermal origin (Figure 2B).

Together these studies show that *LGR5* is expressed, to varying degrees, by established ES tumors and cell lines. Moreover, they demonstrate that while *LGR5* is not highly expressed by MSC of mesodermal origin, it is expressed by neural crest-derived stem cells, in particular undifferentiated NCSC.

### *LGR5* expression is elevated in clinically aggressive ES and in putative ES cancer stem cells

**Figure 3:**
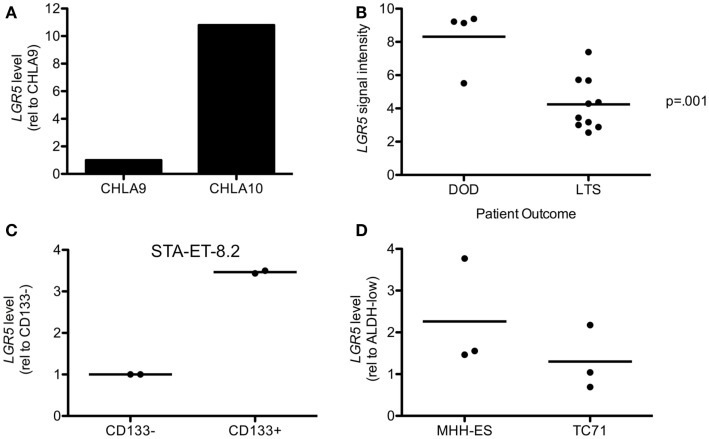
**Leucine-rich repeat-containing G-protein coupled receptor 5 is increased in aggressive disease and putative cancer stem cells**. **(A)** Metastatic tumor-derived CHLA10 cells express higher levels of *LGR5* than CHLA9 cells, which were derived from the primary tumor at diagnosis. **(B)** *LGR5* expression was found by microarray analysis to be increased in tumors from 4 patients with rapidly progressive and fatal primary ES (DOD-dead of disease) compared to 10 patients with at least 48 months disease free survival (LTS-long term survivors). **(C)** *LGR5* levels are higher in CD133^+^ compared to CD133^−^ STA-ET-8.2 cells. Data from two independent sorting experiments are shown and expression on the *y*-axis is of CD133^+^ cells relative to the corresponding CD133^−^ cells. **(D)** *LGR5* expression is increased in ALDH^high^ compared to ALDH^low^ MHH-ES and TC71 cells. Data from three independent sorts are shown and expression on the *y*-axis is that of ALDH^high^ relative to the corresponding ALDH^low^ cells. The horizontal line in **(B–D)** all represent arithmetic mean values.

Over-expression of *LGR5* has been linked to clinically aggressive disease in colorectal carcinoma as well as several other epithelial malignancies. Therefore, we sought to determine if high expression of *LGR5* might also be associated with aggressive disease in ES. To address this we first compared *LGR5* expression in the CHLA9 and CHLA10 cell lines, which were derived from the same patient prior to (CHLA9) and after (CHLA10) chemotherapy. It is noteworthy that the CHLA10 cell line was derived from a metastatic focus and therefore represents a progressive state of disease. As shown, *LGR5* levels were found to be 10-fold higher in CHLA10 than CHLA9 cells (Figure 3A). To evaluate whether clinically aggressive ES express higher levels of *LGR5* *in vivo* we interrogated whole genome expression data that were generated from primary human tumor samples (ERL unpublished data, kindly provided by the COG). *LGR5* expression levels from diagnostic biopsies were compared in 4 patients who succumbed to rapidly progressive disease (survival 5–13 months) and 10 patients who remained disease free for at least 48 months. *LGR5* expression was found to be extremely high in three of four patients with aggressive disease and mean expression was significantly higher than in long term event free survivors (Figure 3B). Interestingly, two of the three patients with the highest expression of *LGR5* had primary drug-resistant tumors.

It has been previously reported that *LGR5* expression is enriched in cancer cells with stem cell-like properties (Merlos-Suarez et al., 2011; Kemper et al., 2012; Kobayashi et al., 2012; Nakata et al., 2013). These cells have the unique ability to self-renew and differentiate compared to bulk tumor cells and can contribute to metastasis and drug resistance (Visvader and Lindeman, 2012). Although putative CSC were identified in a small cohort of primary tumors (Suva et al., 2009) it has been challenging to isolate putative CSC from established ES cell lines. Nevertheless, CD133 surface expression (Jiang et al., 2010) and high-level of ALDH activity (Awad et al., 2010) have been used to successfully enrich for CSC populations in STA-ET-8.2 and MHH-ES and TC71 cells, respectively. Therefore, we used these previously reported ES cell lines and CSC-enrichment assays to determine whether *LGR5* expression is up regulated in putative CSC. qRT-PCR analysis of CD133-sorted STA-ET-8.2 populations consistently demonstrated increased expression of *LGR5* in the CSC-enriched CD133^+^ fraction (Figure 3C). Similarly, levels of *LGR5* were reproducibly higher in MHH-ES cells that displayed high ALDH activity than MHH-ES cells with low ALDH activity (Figure 3D). In contrast, data from TC71 were inconsistent. Although increased expression of *LGR5* was detected in ALDH^high^ CSC populations in one experiment this finding was not reproducible in two other independent experiments (Figure 3D).

In summary, these findings suggest that *LGR5* is highly expressed by at least some populations of CSC-like ES cells. In addition, they provide preliminary evidence in support of the hypothesis that over-expression of *LGR5* in primary tumors may be associated with an aggressive drug-resistant clinical phenotype. Studies are ongoing to determine if this association can be validated in a larger cohort of tumors.

### R-spondin potentiates Wnt/β-catenin signaling in an *LGR5-*dependent manner

**Figure 4:**
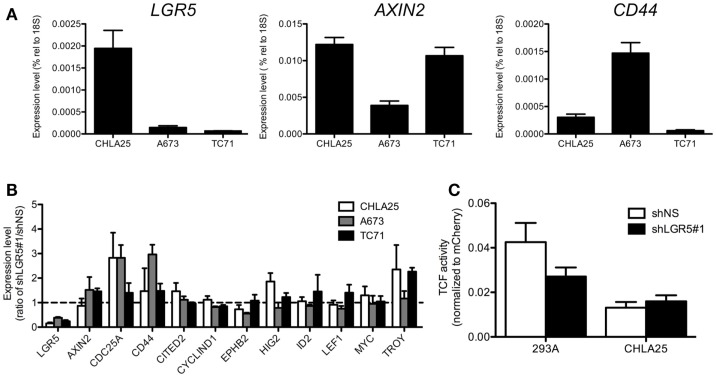
**No correlation exists between *LGR5* expression and Wnt/β-catenin activity in ES cells in standard culture**. **(A)** Canonical Wnt/β-catenin target gene expression was measured by qRT-PCR in ES cell lines. There was no apparent correlation between *LGR5* expression and Wnt/β-catenin target genes *AXIN2* and *CD44*. **(B)** No consistent or significant change occurs in the expression of a panel of Wnt/β-catenin target genes following *LGR5* knockdown. The dotted line represents a ratio of 1 between shLGR5#1 and shNS cells. **(C)** *LGR5* knockdown does not measurably decrease Wnt/β-catenin transcriptional activity. Luciferase activity was determined using TCF reporter-transduced cell lines as described in Section “Material and Methods.” All data shown is from three independent experiments and error bars are SEM.

Given its role as a potentiator of Wnt/β-catenin signaling, we hypothesized that ES cells with high levels of *LGR5* expression would demonstrate relatively high Wnt/β-catenin transcriptional activity. To address this we first evaluated basal levels of the Wnt/β-catenin axis in ES cells that were grown in standard tissue culture conditions. To begin we measured the expression of known Wnt/β-catenin target genes in three different ES cell lines (A673, CHLA25, and TC71) and discovered that although the levels of gene expression varied significantly among the three cell lines, there was no correlation with *LGR5* levels (Figure 4A). In support of this, we observed no significant or reproducible change in the expression of Wnt/β-catenin target genes following *LGR5* knockdown (Figure 4B). In particular, loss of *LGR5* did not result in down-regulation of Wnt targets suggesting that, in the context of standard tissue culture (i.e., in the absence of exogenous RSPO), LGR5 has little impact on Wnt/β-catenin transcriptional activity (Figure 4B). Next, we used TCF-promoter luciferase-reporter assays to directly measure the level of TCF transcriptional activity in CHLA25 ES cells before and after *LGR5* knockdown. These assays confirmed that *LGR5* knockdown had only minimal impact on basal Wnt/β-catenin transcriptional activity in standard culture conditions (Figure 4C). Thus, in standard culture conditions ES cells show only minimal canonical Wnt activity and this activity is not impacted by LGR5.

**Figure 5:**
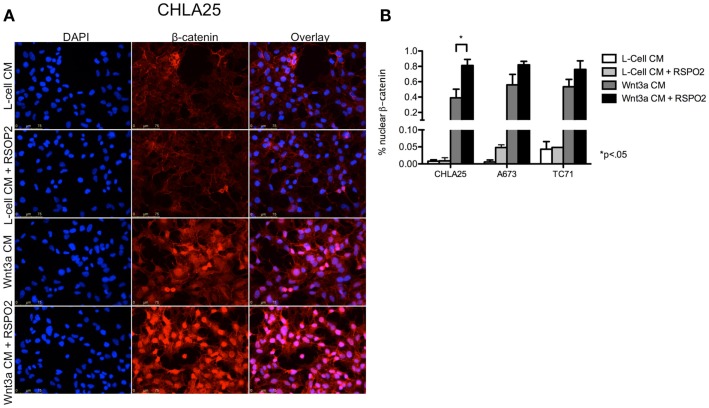
**Exogenous Wnt3a induces nuclear localization of β-catenin and is potentiated by RSPO2**. **(A)** CHLA25 cells grown in the presence of Wnt3a conditioned medium (CM) induced nuclear localization of β-catenin and this was robustly potentiated by the addition of RSPO2. RSPO2 alone had no effect on β-catenin nuclear localization. **(B)** The percentage of CHLA25, A673, and TC71 cells with nuclear β-catenin all increased with Wnt3a CM, but CHLA25 showed the greatest increase in nuclear localization with the addition of RSPO2. Data from three independent experiments and error bars are SEM.

**Figure 6:**
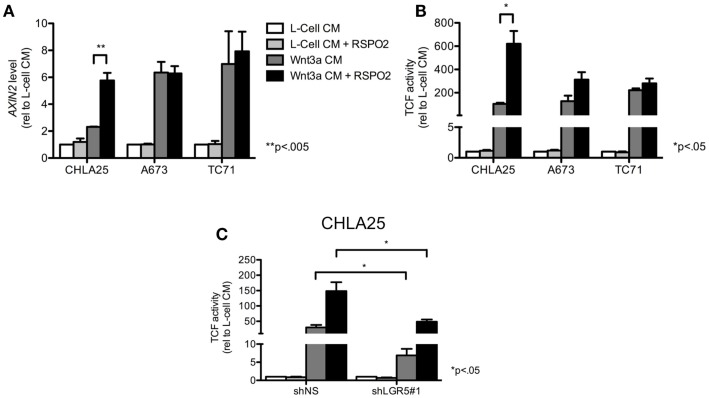
**RSPO2 potentiates Wnt/β-catenin signaling in *LGR5*-high ES cells**. **(A)** Exposure of ES cell lines to Wnt3a conditioned medium (CM) increased *AXIN2* expression, but expression was only potentiated by RSPO2 in the *LGR5*-high cell line, CHLA25. **(B)** TCF reporter activity was induced in ES cells exposed to Wnt3a CM, but was only potentiated in CHLA25 cells by the addition of RSPO2. **(C)** TCF reporter activity was measured in control and *LGR5* knockdown CHLA25 cells following exposure to Wnt3a with/without RSPO2. Reporter activity in the presence of RSPO2 was significantly diminished in *LGR5* knockdown cells. All data shown is from three independent experiments and error bars are SEM.

Next, we investigated whether the Wnt/β-catenin axis is intact in ES cells and capable of activation in the presence of exogenous ligands. In particular, we hypothesized that cells with high levels of endogenous *LGR5* expression might be susceptible to RSPO-mediated potentiation of Wnt/β-catenin signaling. To address this we first evaluated sub-cellular localization of β-catenin in ES cells before and after exposure to exogenous Wnt3a. Consistent with a previous report (Uren et al., 2004), ES cells demonstrated little evidence of β-catenin in their nuclei under basal conditions (Figure 5A). In contrast, exposure of ES cells to Wnt3a CM resulted in robust nuclear translocation of β-catenin (Figures 5A,B). Indeed, Wnt3a induced nuclear translocation of β-catenin in all three lines irrespective of their endogenous *LGR5* expression levels (Figure 5B). Although there are four RSPOs, RSPO2 has been shown to have the highest affinity for both LGR4 and LGR5 (Carmon et al., 2011). In addition, RSPO2 is highly expressed in developing bone (Nam et al., 2007; Hankenson et al., 2010). Therefore, we next exposed ES cells to RSPO2 either alone or in combination with Wnt3a and measured β-catenin nuclear localization. The presence of RSPO2 alone had no effect on sub-cellular localization of β-catenin (Figures 5A,B). In contrast, the combination of Wnt3a CM plus RSPO2 resulted in potentiation of Wnt/β-catenin signaling in CHLA25 cells as demonstrated by further up-regulation and nuclear translocation of β-catenin (Figures 5A,B). We also measured the expression of the Wnt/β-catenin target gene *AXIN2* in these four conditions. In keeping with increased nuclear localization of β-catenin, we observed induction of *AXIN2* expression following Wnt3a stimulation and, in CHLA25 cells, potentiation of this up-regulation in the presence of RSPO2 (Figure 6A). Likewise, TCF reporter activity increased in a stepwise fashion when cells were exposed first to Wnt3a alone and then both Wnt3a and RSPO2 together (Figure 6B). Notably, potentiation of Wnt/β-catenin signaling by RSPO2 was only statistically significant in CHLA25 cells, the cell line with the highest level of *LGR5*. To determine if LGR5 was responsible for mediating this robust RSPO2-dependent potentiation of Wnt/β-catenin signaling we evaluated the consequences of *LGR5* knockdown on this signaling axis in CHLA25 cells. Significantly, in the context of Wnt3a and RSPO2 ligands, knockdown of *LGR5* resulted in reduced potentiation of Wnt/β-catenin transcriptional activity (Figure 6C). Interestingly, we also observed down-regulation of TCF reporter activity in Wnt3a-only treated cells (Figure 6C) suggesting that low level endogenous production of RSPO by CHLA25 cells might also contribute to potentiation of the Wnt/β-catenin axis in a Wnt-rich microenvironment.

### *LGR5* does not promote ES cell proliferation *in vitro*

**Figure 7:**
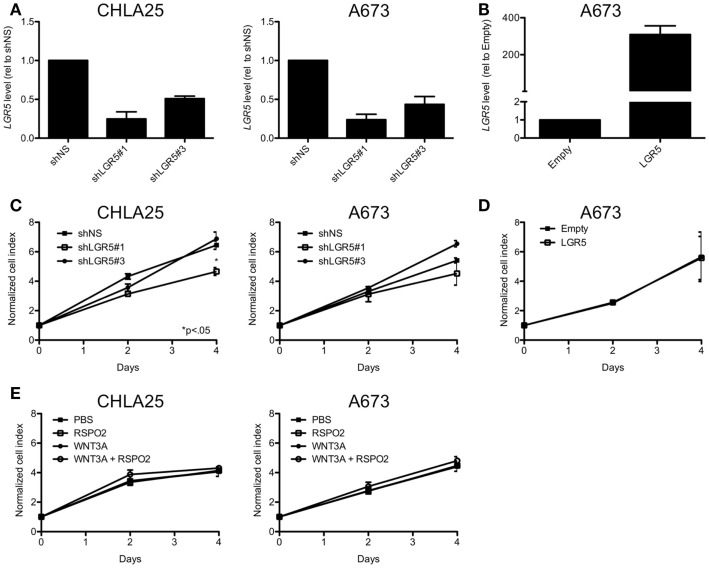
**Leucine-rich repeat-containing G-protein coupled receptor 5 does not promote ES cell proliferation**. **(A)** qRT-PCR confirmation of *LGR5* knockdown in ES cell lines transduced with *LGR5*-targeted shRNAs (shNS = non-silencing control). **(B)** Confirmation of *LGR5* over-expression in A673 cells transduced with *LGR5* over-expression construct (Empty, empty vector control). **(C)** ES cell expansion was measured by MTS assay and was not affected by *LGR5* knockdown. **(D)** *LGR5* over-expression did affect cell growth in A673 cells. **(E)** Parental cell growth was not affected by the addition of WNT3A and/or RSPO2 to standard culture conditions. All data shown is from two independent experiments and error bars are SEM.

Leucine-rich repeat-containing G-protein coupled receptor 5 functions as a growth-promoting oncogene in several adult malignancies including gastrointestinal tumors and gliomas (McClanahan et al., 2006; Tanese et al., 2008; Nakata et al., 2013). In addition, *LGR5* was recently shown to promote proliferation of neuroblastoma (Balamuth et al., 2010), another highly aggressive pediatric tumor of neural crest origin (Jiang et al., 2011). In order to determine if *LGR5* promotes ES cell proliferation we generated ES cell lines with altered levels of expression of *LGR5* as a consequence of stable RNA-interference-mediated knockdown or ectopic over-expression (Figures 7A,B). As shown, although reduced proliferation of the CHLA25 cell line was observed with one *LGR5*-targeted knockdown sequence (shLGR5#1), this phenotype was not observed in A673 cells (Figure 7C). In addition, a second *LGR5*-targeted shRNA sequence (shLGR5#3) did not inhibit proliferation in any of the cell lines (Figure 7C). Forced over-expression of *LGR5* in the *LGR5-* low cell line A673 also had no impact on proliferation (Figure 7D). Cellular viability was also measured by trypan blue staining and no change was observed in any of the cell lines following *LGR5* knockdown or over-expression (data not shown). Therefore, in contrast to neuroblastoma cells (Balamuth et al., 2010), *LGR5* does not promote the proliferation of ES cells in standard *in vitro* culture conditions.

Next, we repeated proliferation studies in non-transduced, parent cells in the presence of WNT and RSPO, reasoning that changes in proliferation downstream of Wnt/β-catenin activation would only be apparent in cells that were exposed to the necessary ligands. Interestingly, no change in proliferation was observed in either A673 or CHLA25 cells with the addition of WNT3A and/or RSPO2 (Figure 7E). Studies are ongoing to evaluate the phenotypic consequences of LGR5-mediated potentiation of Wnt/β-catenin signaling but these data support prior observations that this pathway does not promote ES cell proliferation (Endo et al., 2008).

In summary, these studies demonstrate that the WNT/β-catenin signaling axis is present and intact in ES cells but is not activated in standard cell culture conditions. Addition of Wnt ligand to ES cell culture media can activate the canonical pathway and, in CHLA25 cells that express high levels of *LGR5*, activation is potentiated by the addition of RSPO2. The generalizability of this observation to other LGR5 over-expressing ES cells and primary tumors now requires further investigation to fully elucidate the contribution of the LGR5-WNT/β-catenin signaling axis to ES pathogenesis.

## Discussion

In this study we have, for the first time, shown that *LGR5* is expressed by ES cells, both in the context of primary tumors and in cell culture, but that expression levels vary significantly among different ES cell populations. In particular, we found that levels of *LGR5* were highest in putative ES cancer stem cells as well as primary tumor biopsies obtained from patients with rapidly progressive and drug-resistant disease. The wide range and pattern of *LGR5* expression in ES tumor samples and cell lines is consistent with patterns of *LGR5* expression that have been discovered in other human cancers. Specifically, recent studies of colorectal carcinoma support the hypothesis that LGR5 is a marker of cancer stem cells in these tumors (Kemper et al., 2012; Kobayashi et al., 2012). In addition, studies of colorectal and gastric carcinoma, glioblastoma, and esophageal adenocarcinoma have all demonstrated heterogeneity of *LGR5* expression and shown that high *LGR5* levels are associated with worse outcomes (Becker et al., 2010; Simon et al., 2012; Wu et al., 2012; Nakata et al., 2013). Likewise, several studies have found a connection between high *LGR5* expression and chemoresistance (Bauer et al., 2012) and metastasis (Uchida et al., 2010; Takahashi et al., 2011; Valladares-Ayerbes et al., 2012; Wu et al., 2012) in gastrointestinal malignancies. Interestingly, recent studies in mouse models of neuroblastoma (Balamuth et al., 2010) and medulloblastoma (Kawauchi et al., 2012) also discovered increased expression of *Lgr5* in the most aggressive tumors. Thus, there is now substantial evidence in tumors of both epithelial and neural origin to implicate LGR5 as a marker of an aggressive cellular phenotype. Data to support a potential role for LGR5 in tumors of mesenchymal origin is also now beginning to emerge. A recent report by Rot et al. (2011) described a novel splice variant of *LGR5* in the context of soft tissue sarcoma and reported that low level expression of this variant transcript (which lacks exon 5) was associated with worse overall and event free survival. In addition, the *LGR5* locus was recently identified to be among the most frequently amplified loci in a genome-wide study of soft tissue sarcomas (copy number in top 1% of nearly 19,000 genes) suggesting that up-regulation of *LGR5* may contribute to sarcoma pathogenesis (Barretina et al., 2010). Our current study adds ES to the list of tumors where *LGR5* may serve as a marker of aggressive disease. We are now actively pursuing this hypothesis in large cohorts of primary tumor samples as well as model systems.

Although LGR5 was identified some years ago as a Wnt-responsive gene, its role as a potentiating receptor upstream of Wnt signaling was only recently discovered (Carmon et al., 2011; de Lau et al., 2011; Glinka et al., 2011; Gong et al., 2012). LGR5 and its related receptor family members LGR4 and LGR6 have now all been established as a receptors for the RSPO-family of ligands, which act to potentiate Wnt/β-catenin signaling by complexing with Frizzled/LRP receptors (Carmon et al., 2011; de Lau et al., 2011; Glinka et al., 2011; Gong et al., 2012). There is now substantial evidence that hijacking of Wnt signaling may be important for the initiation and maintenance of ES, although the precise contribution of β-catenin-dependent and – independent pathways remains to be elucidated (Uren et al., 2004; Miyagawa et al., 2009; Navarro et al., 2010; Vijayakumar et al., 2011; Hauer et al., 2013). Significantly, Uren et al. (2004) measured expression of the canonical Wnt ligands *WNT1*, *WNT2*, and *WNT3* and found them to be undetectable in ES. In addition, only low levels of β-catenin nuclear localization were detected in cell lines and tumor samples. Likewise, Navarro et al. (2010) reported low levels of basal TCF transcriptional activity in ES cell lines. Studies of DKK1 and DKK2, known modulators of Wnt signaling, have demonstrated that while the former is repressed in ES (Navarro et al., 2010) the latter is induced by EWS-FLI1 and promotes cellular invasion and metastasis (Miyagawa et al., 2009; Hauer et al., 2013). In addition, a recent report showed that the non-canonical Wnt, WNT5A, induces ES cell migration and that the Wnt inhibitor SFRP5 is silenced in ES cells by DNA promoter methylation (Jin et al., 2012). Thus, although there is still much to be understood about the role of Wnt signaling in ES pathogenesis, the data thus far support a more dominant role for Wnt deregulation in altering cell morphology, motility, and invasion rather than classical β-catenin-mediated cellular proliferation. Consistent with these prior reports we found that, in standard culture conditions, basal levels of Wnt/β-catenin signaling are low. In addition, despite the fact that Wnt3a and RSPO2 were able to robustly activate and potentiate canonical Wnt signaling, respectively, we did not observe changes in cellular proliferation. Even the addition of RSPO2 to the culture media of *LGR5-*high CHLA25 cells had no impact on cell proliferation. Likewise, *LGR5* knockdown had no discernible impact on the expression of Wnt/β-catenin target gene expression nor TCF reporter activity under basal conditions. However, when growth medium was supplemented with Wnt3a, β-catenin nuclear localization, and TCF transcriptional activity were robustly activated. In ES cells that express *LGR5* this signaling was further potentiated by the addition of RSPO2 confirming a functional role for this receptor in cells that are exposed to LGR5 ligand. Thus, our results together demonstrate that, in the presence of appropriate ligands, Wnt/β-catenin signaling can be stimulated in ES cells and this stimulation is maximal in cells that express high levels of *LGR5*. The phenotypic effects of this increased Wnt activity remain unclear but our studies thus far suggest that proliferation is unaffected and that both cell autonomous as well microenvironmental factors combine to determine the functional consequences of enhanced Wnt signaling and nuclear β-catenin localization in LGR5^+^ ES cells.

Emerging evidence from other model systems supports the possibility that LGR5 function might be highly contextually dependent. Mouse studies have shown that *Lgr5* is widely expressed during embryogenesis but only becomes limited to discrete stem cell populations post-natally, in particular stem cells of hair follicles and the gastrointestinal tract (Barker et al., 2007; Barker and Clevers, 2010). In normal stem cells secretion of RSPO from neighboring cells supports *Lgr5-*dependent self-renewal and proliferation by activating canonical Wnt signaling (Barker and Clevers, 2010). Likewise, the initiation and proliferation of intestinal adenomas and carcinomas is driven by Lgr5^+^ stem cells (Barker et al., 2009; Schepers et al., 2012). However, data from both colorectal carcinoma cell lines as well as other tumor types complicate the scenario. Consistent with our own data, Walker et al. (2011) found that LGR5 did not affect proliferation of colorectal cancer cell lines but instead negatively regulated Wnt signaling, colony formation, and migration. Thus, in this cellular context LGR5 functioned more as a tumor suppressor than tumor promoter. In addition, promoter hypermethylation and loss of function mutations in *LGR5* and *LGR6* have been discovered in some colorectal tumor samples, again suggesting that these genes could act as tumor suppressors in some contexts (Sjoblom et al., 2006; Chan et al., 2008; de Sousa et al., 2011). These conflicting reports demonstrate that *LGR5* has differing roles during development and in different cellular contexts. Thus, its contribution to cancer maintenance and progression is likely to be determined by both the genetic and epigenetic state of the affected cell as well as the surrounding microenvironment.

The mechanism of transcriptional up-regulation of *LGR5* in ES cell populations is unknown. Importantly, however, *LGR5* is not induced by EWS-FLI1 and unpublished data from our own lab as well a previously published report (see Navarro et al., 2010 supplementary data) suggest that *LGR5* is, in fact, repressed by EWS-FLI1. Thus, we reasoned that expression of *LGR5* in ES cells may instead be a reflection of their putative stem cell origins. Interestingly, among different neuro-mesenchymal stem cell populations we discovered that undifferentiated NCSC expressed the highest levels of *LGR5*. Expression was still detectable, albeit at lower levels, in NCSC that had undergone epithelial-mesenchymal transition to an MSC-state (von Levetzow et al., 2011). In contrast, bone marrow-derived MSC did not express detectable levels of *LGR5*. Intriguingly, it is now established that rare MSC in the bone marrow are derived from the neural crest (Takashima et al., 2007; Nagoshi et al., 2008) and gene expression profiling data from mouse MSC (GEO accession GSE30419) showed increased expression of *Lgr5* in neural crest-compared to mesoderm-derived MSC populations (Wislet-Gendebien et al., 2012). Based on these studies we now speculate that at least some ES might arise from neural crest-derived LGR5^+^ stem cells in the bone marrow. Further, we speculate that the RSPO-rich microenvironment of developing bone may contribute to malignant transformation of these LGR5^+^ cells in the event that they acquire an EWS-ETS fusion. Ongoing studies in our laboratory are now specifically addressing these intriguing hypotheses.

In summary, we have shown that *LGR5* is expressed by ES, in particular by putative cancer stem cells and, in the context of a Wnt and RSPO-rich microenvironment, LGR5 functions to potentiate canonical Wnt/β-catenin signaling. In addition, we have discovered that *LGR5* is expressed by neural crest-derived stem cells, putative cells of ES origin, demonstrating that LGR5 may also be a marker of some non-epithelial stem cells. Given the profound complexity of Wnt signaling and its dependence on both cell autonomous as well as microenvironmental cues it is now essential that functional studies of LGR5 and the Wnt/β-catenin axis in ES be performed in model systems that faithfully recapitulate the *in vivo* tumor microenvironment.

## Conflict of Interest Statement

The authors declare that the research was conducted in the absence of any commercial or financial relationships that could be construed as a potential conflict of interest.
